# The Emergence of Risk Communication Networks and the Development of Citizen Health-Related Behaviors during the COVID-19 Pandemic: Social Selection and Contagion Processes

**DOI:** 10.3390/ijerph17114148

**Published:** 2020-06-10

**Authors:** Seunghoo Lim, Hiromi Nakazato

**Affiliations:** 1 Public Management and Policy Analysis Program, Graduate School of International Relations, International University of Japan, Niigata 949 7277, Japan; 2 School of Information and Communication, Meiji University, Tokyo 101 8301, Japan; nakazato@meiji.ac.jp

**Keywords:** risk communication networks, citizen behaviors, voluntary public health measures, health resilience, COVID-19, coevolution, stochastic actor-oriented model

## Abstract

Amid the novel coronavirus pandemic, a variety of public health strategies have been implemented by governments worldwide. However, the fact that strict government mandates focus on physical distancing does not mean that social connectedness for voluntary risk communication among citizens should be sacrificed. Furthermore, we lack an understanding of citizens’ behaviors regarding the voluntary adoption of public health measures and the control of mental wellbeing in the age of physical distancing. Key variables in the response to the global pandemic are the emergence of risk deliberation networks, voluntary compliance with government guidelines, and the restoration of citizens’ subjective health. However, little is known about how citizens’ health-related behaviors coevolve with social connections for sharing information and discussing urgent pandemic issues. The findings show that selection and social influence mechanisms coexist by affecting each citizen’s health-related behaviors and community-led risk discourses in the face of the urgent health crisis.

## 1. Introduction

As of 18 April 2020, the novel coronavirus (COVID-19) had killed 146,088 and infected 2,160,207 people globally [1]. Worldwide, governments at multiple levels, including the central, provincial, and municipal levels, are seeking to contain the spread and outbreak of COVID-19. However, discussions surrounding COVID-19 have not paid sufficient attention to the significance and role of risk communication that voluntarily emerges in civil society [2], despite the strong evidence from the field of risk governance that discourses on risk impact disaster response and community resilience during shocks [3]. Furthermore, there is much controversy that surrounds how much governments should intervene in individuals’ freedom and enforce changes in their behaviors [4], and there is considerable discussion of how we can promote citizens’ voluntary compliance with government policies or mandates to change their behaviors across countries [5,6]. At the same time, citizens other than those in vulnerable groups have become anxious about their deteriorating mental health caused by sudden and prolonged isolation from society beyond the current infection issues [7,8]. The discussion within societies and governments regarding regulatory interventions mainly calls for and emphasizes physical distancing—for example, enforcements to impose lockdowns, shut down shops, or restrict the movement of citizens—and the effectiveness of these measures to prevent COVID-19. The social connections for sharing critical information and exchanging mental health support have been neglected or ignored. This paper aims to investigate how the emergence of social connectedness through or for risk information-sharing in a community amid the global pandemic is related to: (i) citizens’ voluntary adoption of public health measures recommended by the government to maintain a safe physical distance and decrease transmission, and (ii) citizens’ mental well-being in the age of physical distancing, and vice versa, over time.

## 2. A Theoretical Conjecture on the Dynamics between Risk Communication Networks and Health-Related Behavioral Changes in Pandemic Settings 

Risk communication can be defined as the interactive process of exchanging information and opinions among individuals, groups, and any stakeholders in a community concerning an emergent or potential risk to human health or the environment to make better decisions about their well-being during the crisis [3,9,10,11]. The importance of risk communication for developing health resilience and improving COVID-19 responses in communities has drawn enormous attention [12,13]. The positive relationships between risk communication and the health resilience of citizens in pandemics have been reported in various recent studies on disasters, health, and communities [14,15,16]. However, the previous research—which is mostly based on cross-sectional data collected at only a single time point—is limited in its empirical examination of: (i) whether citizens’ health behaviors (such as their adherence to voluntary public health measures and their subjective health, as examined in this paper) affect the formation of risk deliberation networks among them; (ii) whether citizens’ health-related behaviors can be adjusted by risk communication interactions in their communities; and (iii) which causal mechanism between (i) and (ii) is a stronger trigger over time. Accordingly, it is challenging to identify and determine multiple competing causal links and examine their distinct effects without longitudinal data collected at several separate time points.

Several concepts have been developed in sociology [17] but are not fully utilized in risk governance and communication. These concepts including selection (or homophily) versus contagion (or influence) and the social network analysis technique for the coevolutionary process between network formation and behavioral changes [18], which will be discussed later, allow us to tackle the theoretical and empirical aspects of the longitudinal mutual interactions between the emergence of health communication and the progress of health-related behaviors. Selection (or homophily) takes place when the formation of ties is initiated based on similar individual attributes or the same characteristics between actors [19]. In contrast, contagion (or influence) occurs when ties between actors are first developed, and then, their distinct individual properties are mutually and gradually adapted to each other via the previously established links [20,21]. As mentioned above, multiple causal mechanisms compete to explain: (i) the development, maintenance, or termination of a tie in any pair of two actors in a risk communication network (i.e., selection mechanism); and (ii) the health-related behavioral changes that occur through each actor’s connections with other people when deliberating about health risk (i.e., influence or mutual adaptation mechanism) [22].

### 2.1. Risk Communication Network Dynamics

#### 2.1.1. Health-Related Behavior Mechanism (the Selection Effect in the Coevolutionary Process)

We first conjecture that citizens’ health-related behaviors during the COVID-19 pandemic are a critical impetus for the formation of ties for risk discourses. The three principal mechanisms that drive the search for risk communication described below—including: (i) the eligibility and entitlement mechanisms [18], and (ii) the homophily mechanism [19]—attribute the establishment of the links for risk information-sharing to citizens’ distinct levels of health-related behaviors, such as adopting voluntary preventive health measures and changing their subjective health during this critical time of the COVID-19 pandemic.

#### Eligibility for and Entitlement to Risk Communication

Citizens who have already adopted voluntary public health measures or perceive their subjective health as good tend to be recognized by other people as sources of information regarding the current health risk issues and are more easily engaged in risk communication [23,24]. In a pandemic situation, citizens who have adopted more preventive health measures or have high subjective health have become pivotal community resources who are approached by other people to expedite health communication. The better risk preparedness/awareness or better subjective health of these citizens also stimulates them to respond to other people’s need to communicate [25]. In contrast, individuals who have adopted fewer public health measures or assess their subjective health as lower are more likely to contact other citizens and urgently seek communication about the current risks [26,27,28]. During a pandemic period, individuals with lower risk preparedness/awareness or with worse self-assessed health conditions such as stress, depression, or mental disorders are entitled to seek information from other people and legitimize their higher frequency of approaching others for risk communication.

#### Homophily

Another mechanism of the evolution of pandemic communication networks attributes the formation of communicative ties to similarities in the adoption of voluntary public health measures or the degrees of subjective health perceived by any pair of two citizens within a specific risk communication network. During a pandemic, if such homophily (that is, an effect of the similarity of voluntary public health measures or subjective health on the formation of risk information-sharing ties) exists [21,29], connections for discussing health risk among individuals will be promoted by their similar health-related behaviors, that is, either a higher adoption of voluntary public health measures or higher subjective health ratings. Of course, the opposite types of homophilic connections for risk communication are also expected to emerge between individuals with a lower adoption of voluntary public health measures or lower subjective health. Homophilic partners who seek risk discourses with actors based on similar levels of health-related behaviors during a health emergency [30] indicate that citizens with better preparedness, awareness, or subjective health do not communicate with other people who are less prepared, adopt fewer preventive measures or have poor subjective health. In contrast, individuals with lower risk preparedness/awareness or poor subjective health may be hesitant to communicate with better prepared or mentally healthy partners and tend to approach other people with similar vulnerability for risk communication. These probable patterns of preferential partner selection could lead to segregation by health risk preparedness or subjective health and discrimination against more vulnerable groups based on a low adoption of public health measures or poor subjective health in a pandemic. This indicates a potential dark side of risk communication in health emergency settings [29].

### 2.2. Health-Related Behavior Dynamics

#### 2.2.1. Structural Mechanisms (the Degree Effect in the Coevolutionary Process)

The selection mechanisms of the formation of risk deliberation networks mentioned above—according to which citizens’ adoption of voluntary public health measures and self-reported subjective health affect their networking activities for pandemic communication—explain only one direction of the mutual interactions between these two variables. Thus, we also need to contemplate the other direction, i.e., the reverse effect of forging links for risk communication on shifts in the voluntary adoption of public health measures or subjective health. The three mechanisms—including approaching other people, being approached by other people, and mutual adaptation [18]—propose that the voluntary adoption of public health measures and the self-rated health of individuals are dependent on the connections between them to communicate about and discuss emerging and urgent health concerns and risks.

##### Approaching Other People

When individuals can more frequently approach other people to discuss the health risk issues regarding COVID-19 and are thus more easily able to obtain necessary information, they will adopt more voluntary countermeasures or perceive their subjective health statuses more positively. More opportunities or chances for people to seek deliberation about risk with other individuals implies an increasing likelihood to deliberate on the utilities or significance of voluntary public health measures or evaluate their subjective health statuses to be more resilient and to bounce back from worries, concerns, or mental disorders during the pandemic, as they can more easily have dialogues with other people and obtain required information from them [26,31]. In our case, as citizens can frequently contact other people through offline or online channels to obtain the information that they need during this pandemic—which is measured by each focal citizen’s out-degree ties—they are more likely to learn about and share how to prevent potential health risks or to perceive that they are able to overcome present obstacles and improve their subjective health to endure possible risky situations through discussions about this urgent issue [32,33].

##### Being Approached by Other People

The previously described mechanism of health-related behavioral changes, i.e., approaching other people, suggests that individuals’ initiatives and willingness to form links to other people to gain information about and discuss current health emergency issues may influence their voluntary adoption of public health measures or their perceived subjective health during the COVID-19 pandemic. In addition, their voluntary countermeasures and self-rated subjective health statuses could be influenced by how often they are approached or contacted by other individuals for risk information-sharing [34]. That is, when citizens are more frequently approached by other people for communication and are asked to share information or knowledge, they will recognize that other individuals view them as appropriate and timely sources of information who are essential in the pandemic. This perception of their contribution to or utility in their community will motivate them to take more countermeasures for their community and positively affect their perceived level of health [32,33,35]. In this context, each focal citizen’s in-degree ties—which demonstrate how other people approach or contact the focal citizen for risk deliberation—will positively affect this citizen’s voluntary adoption of public health measures or his or her subjective health.

#### 2.2.2. Associational Mechanisms (the Mutual Adaption/Influence Effect in the Coevolutionary Process)

In the former two mechanisms of the influence of the ties for risk communication that focal citizens forge or receive on their adoption of voluntary public health measures or self-evaluated health, we contemplated only how often or actively citizens pursue connections to other people (i.e., by developing outgoing links) or are connected to other people (i.e., accepting incoming links), regardless of whom the ties are built with and the individual properties by which the partners are tied. Now, we move to the last but critical mechanism regarding the mutual adjustment of the voluntary adoption of public health measures or subjective health perceived by both connected citizens. In a context in which individuals with distinct levels of countermeasures adopted for health protection or self-reported health are linked to each other for health communication during this pandemic, each citizen’s acceptance of public health preventive measures or subjective health may be adapted to and influenced by those of their risk communication partners [36,37,38]. Through a social contagion or reciprocal influence process mediated by repeated connections for discussions on health risk over time among them, citizens’ individual adoption of public health preventive measures or self-evaluation of health may spread through the previously established ties for risk discourses and then lead to the convergence of these properties toward a specific level [21,36].

## 3. Stochastic Actor-Oriented Model and Data

We intend to determine: (i) how citizens’ engagement in deliberation about health risk influences or adapts to their discussion partners’ adoption of voluntary public health measures or levels of subjective health, and (ii) how citizens forge ties for discourses on health risk with other citizens who have voluntarily adopted different levels of preventive health measures or have different subjective health statuses. To distinguish the causal mechanisms of partner selections for risk communication from the influence or contagion of voluntary public health measures or subjective health among the interdependently connected students for risk communication during the COVID-19 pandemic, we use the stochastic actor-oriented model (SAOM) [22,39,40,41]. The core assumptions of SAOM are that: (i) the network panel data are considered to be repeated snapshots of an evolving process over continuous time, and this process follows a Markov process that assumes that the probabilities of tie changes (i.e., future formation or the termination of a single tie) are dependent on the current state of the network; and (ii) these tie changes are determined by the actors [42]. In the current study’s model of the coevolutionary dynamics between network formations and behavioral changes, individual rational actors determine the possibilities of certain modifications to their risk deliberation ties and health-related behaviors to maximize their own utilities by selecting other actors in the network. We specified our SAOM by including the appropriate network and behavior statistics necessary for investigating the abovementioned effects based on the relevant theories and previous literature described in the preceding section. To satisfy this model’s assumption regarding the decomposability of changes in link and behavioral developments, which are the dependent and independent variables in this research, we repeatedly measured these changes from the same set of actors.

In this study, the boundary of networks [43] for risk information-sharing was specified by the same set of actors, i.e., 37 first-year Masters of Public Administration (MPA) program students with diverse backgrounds from 14 countries in a Japanese graduate school located in a small and remote town. All of these MPA students in this English-speaking-only graduate school are foreigners (i.e., non-Japanese) with extremely limited or elementary Japanese proficiency and around seven months of living in Japan. Thus, these international students with language barriers may be more vulnerable during the pandemic in Japanese society and may have a greater demand for appropriate information to cope with the urgent health crisis [44,45]. This set of 37 MPA students as well as neighbors who reside within the same boundaries of the local community—mainly school dormitories on a campus where no COVID-19 case was reported locally—could be regarded as an easily identifiable and distinguishable boundary of a network to examine the mutual influence between emerging pandemic communication and changing health resilience over time in a specific place. Furthermore, the concern about the possibility that university communities during a pandemic could cause cluster infections was growing from late March in a few Japanese prefectures [46].

From these 37 MPA students, longitudinal network data were collected over three sequential time points—between 18 and 27 March 2020, before the serious nationwide outbreak of COVID-19 in Japan and the declaration of a state of emergency by Japanese national and provincial governments. We performed measurements of risk communication, the adoption of voluntary public health measures generally recommended by the Japanese government, and subjective health at three time points. The first survey was conducted between 18 and 21 March (34 responses out of 37), the second survey was conducted between 22 and 24 March (32 responses out of 37), and the final survey was conducted between 25 and 27 March 2020 (31 responses out of 37). This approach allowed us to observe the decomposable multiple mini-steps within the same boundary of a network that comprised 37 actors. More specifically, for these three distinct and subsequent periods, 37 students were asked about: (i) their health risk discussion partners during the COVID-19 pandemic (“With whom did you have discussions or communication about recent COVID-19 issues over the specific time period?”); (ii) the voluntary public health measures that they adopted (including physical distancing, hand-washing, mask-wearing, and stocking up on groceries, medicine, and necessary resources) [47]; and (iii) their current general health perceptions, which were measured by using the 12-item General Health Questionnaire (GHQ-12) [48]. Although the GHQ-12 excludes the items related to physical symptoms from the GHQ-60 due to false-positive responses to these items, the GHQ-12 has been extensively employed and tested as an instrument to measure psychological well-being and mental illness in numerous contexts and countries with results that have a high degree of validity [49,50].

## 4. Results

Table 1 presents the estimated results for the dynamics between the tie formations for risk deliberation and two separate types of health-related behavioral changes (i.e., adopting voluntary public health measures and developing subjective health) over time.

**Figure 1:**
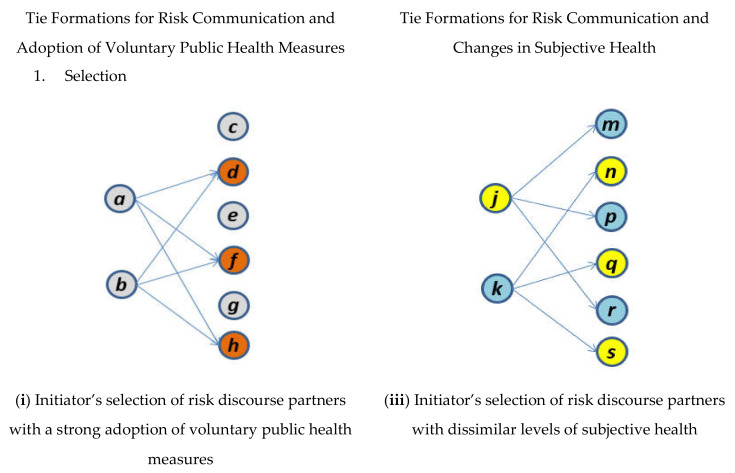

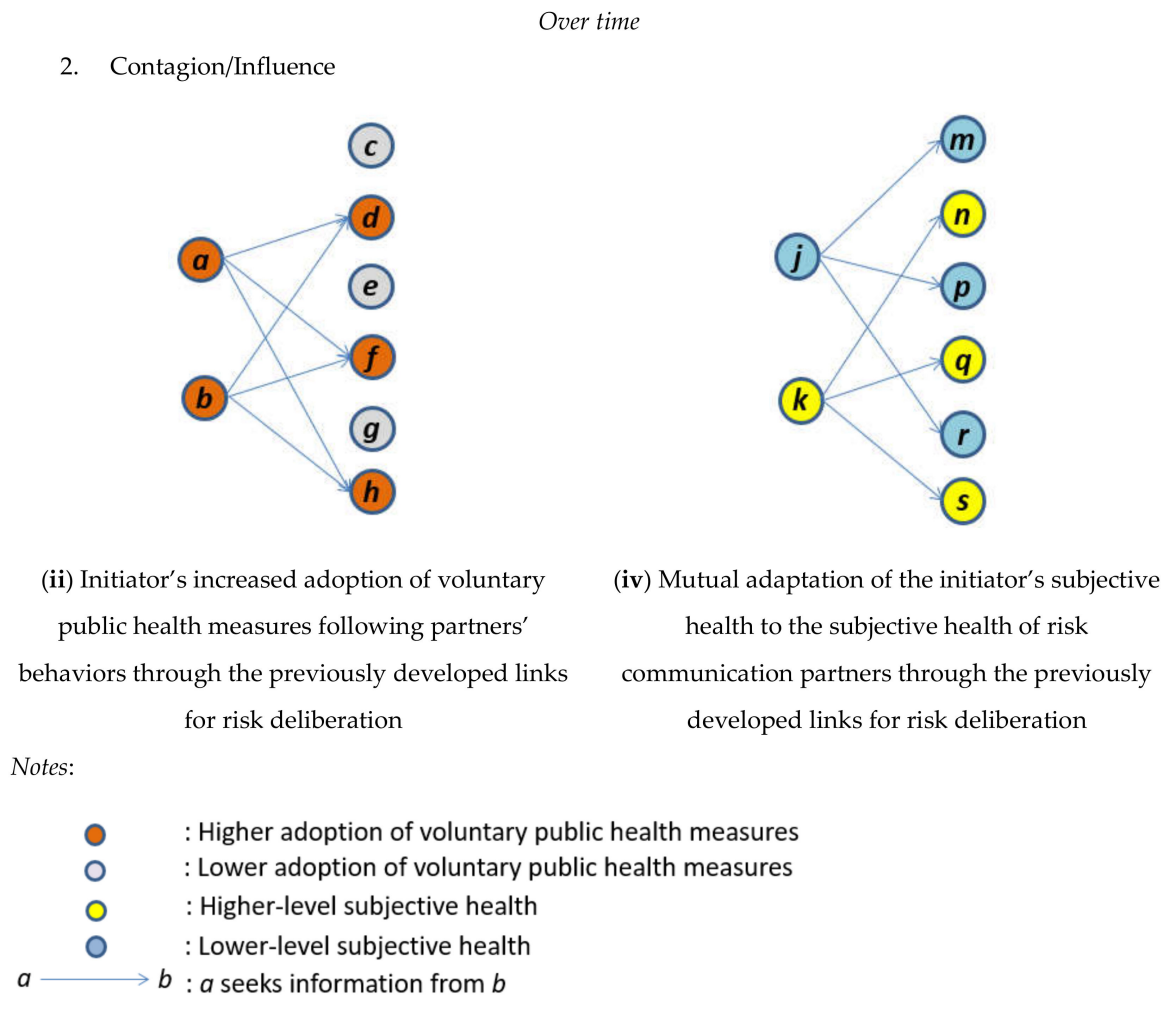
Sequences of Interplay between Risk Communication Networks and Health-related Behaviors over Time.

First, in the model for network dynamics that investigates how health communication ties are formed, the results show that a higher adoption of voluntary public health measures by risk deliberation partners (i.e., alters) facilitates the establishment of links for risk communication by the students (parameter 4: 0.26, *p* < 0.05). That is, the students are more likely to seek information about current health risks from or discuss these issues with other students who have already voluntarily adopted more public health preventive measures. In the model of behavior dynamics that evaluates the determinants of the voluntary adoption of public health measures, mutual influence finds support (parameter 22: 0.35, *p* < 0.05), which indicates that the students’ degrees of the adoption of voluntary public health measures adapt to the degree of the adoption of voluntary public health measures of their risk discourse partners through the connections developed between them. As shown in the left column of Figure 1, these supported effects—eligibility for risk communication (parameter 4) and mutual influence (parameter 22)—sequentially demonstrate the coevolutionary dynamics between the emergence of pandemic communication ties and voluntary public health measure adoption. First, students (*a* or *b*) are likely to approach other students (*d*, *f*, or *h*) who have already adopted more preventive public health measures and to seek information on, or discussion about, the recent pandemic issues from them (see (i) in Figure 1). Then, highly adopted public health measures by such partner students diffuse through the previously established ties for risk discussion, and the adopted voluntary measures for public health of the health communication seekers (*a* or *b*) converge with the adopted voluntary public health measures of their risk deliberation partners (*d*, *f*, or *h*) (see (ii) in Figure 1).

Second, in contrast to our prior expectation of the students’ homophilic tendencies for selecting pandemic communication partners based on similar subjective health, the formation of links for risk communication is driven by heterophily (parameter 9: −1.25, *p* < 0.05). This heterophilic effect regarding the observed subjective health statuses indicates that students with high subjective health pursue discourses about health risk with other students with lower subjective health and vice versa. In the model of self-reported health dynamics that evaluates the determinants of subjective health, only the social contagion and influence effect is empirically supported (parameter 29: 0.65, *p* < 0.10). That is, the students’ self-rated health is mutually adapted to the self-rated health of their discussion partners to whom they are tied for risk communication over time. As shown in the right column of Figure 1, the confirmed heterophilic and mutual influence mechanisms can be combined sequentially to explain the coevolutionary dynamics between the formation of risk information exchange ties and the development of subjective health over time. At first, a student (*k*) with relatively low subjective health tends to approach students (*n*, *q*, and *s*) with better subjective health; in contrast, a student (*j*) with better health is likely to contact students (*m*, *p*, and *r*) with worse health to discuss current issues related to the pandemic (see (iii) in Figure 1). Then, later, the higher subjective health of the risk discourse partners (*n*, *q*, and *s*) are transmitted to and influence the focal approaching student (*k*), who initially had lower health, through the ties formed before for risk discussion, which leads to the convergence of this focal student’s subjective health and the subjective health of his or her risk deliberation partners (i.e., *k*’s improved subjective health condition; see (iv) in Figure 1). In contrast, the lower subjective health ratings by the risk communication partners (*m*, *p*, and *r*) of an approaching student (*j*) with initially higher subjective health diffuse through the previously forged links for health communication, and this focal student’s subjective health decreases toward the health of the risk discourse partners (i.e., *j*’s decreased health; see (iv) in Figure 1).

## 5. Discussion

The abovementioned findings have two implications for public officials, health policy-makers, and risk management practitioners to understand the emergent and underlying mechanisms of risk discourses among citizens and the changes in citizens’ health-related behaviors, to increase citizens’ voluntary compliance with governmental guidelines and to curb the spread or outbreak of the COVID-19 pandemic effectively in a community. First, regarding the dynamics between risk communication and the voluntary adoption of public health measures, the selection mechanism found in this research shows that citizens’ behavior of voluntarily adopting more health preventive measures could work as a signal that shows the severity of the current health emergency to other members of a community and facilitates risk information exchanges and discussion about how to prepare for and tackle the emerging health issues within a community. At the same time, the social influence/contagion mechanism demonstrated in this study suggests that the connections and dialogue for risk information-sharing among citizens who have already adopted voluntary public health measures could function as social pressure on other citizens, and the adoption of preventive public health measures could be developed as a social norm with which they need to comply within a community.

Second, regarding the dynamics between risk communication and subjective health, our analysis has revealed first, the heterophilic selection of risk deliberation partners, which indicates the formation of heterogeneous discussion partners according to citizens’ distinct subjective health. That is, via the heterophily effect, we found not only that subjectively healthier citizens are approached by subjectively less healthy citizens to share information about and discuss COVID-19 issues but also that subjectively less healthy citizens are contacted by subjectively healthier citizens to communicate about the pandemic. The main purposes of citizens with higher self-rated health in seeking discussion and communication about health risks with subjectively less healthy citizens can be understood based on the current inherent contexts and backgrounds of the swift and widespread outbreaks of COVID 19 across communities and regions. That is, effective control of the pandemic can be achieved not only by each citizen’s own effort but also by other citizens’ commitments and community-level efforts to cope with it within a community [51]. Amid the COVID-19 pandemic, the engagement of citizens in health communication networks—regardless of whether citizens rate their subjective health positively or suffer from mental disorders, stress, or worries—is spontaneously emerging in this case. However, combined with the mutual contagion mechanism that leads to the convergence of the levels of subjective health, these citizens’ heterophilic tendencies in selecting risk discourse partners engendered the mixed results. The first positive pattern was characterized by virtuous circles that enhanced the subjective health statuses of all citizens involved in pandemic communications when subjectively less healthy citizens’ concerns and worries about the current pandemic issues were communicated with subjectively healthier citizens. As the approaches for obtaining health risk information by citizens with initially worse subjective health were responded to by citizens with better subjective health, they could obtain the necessary information about the urgent health crisis, perceive connectedness with their community, and promote their subjective health. The second contrasting negative pattern was the vicious circles that lead to the deteriorated subjective health, on average, of risk discourse participants when citizens with better subjective health initiated risk communication with and were tied mostly to citizens with poor subjective health, despite the wider involvement of citizens (regardless of their subjective health statuses) in citizen-oriented, voluntary, spontaneous, and bottom-up risk information exchanges within a community amid the COVID-19 pandemic.

## 6. Conclusions

This empirical study examined which mechanism—selection or social influence—is a more dominant driver of the coevolutionary interactions between the emergence of risk deliberation ties and the development of two separate types of health-related behaviors—namely, adopting voluntary public health measures and changing subjective health—during the COVID-19 pandemic by using SAOM with relational data. The findings observed in this study indicate that the two mechanisms coexist by affecting the health-related behaviors of each member of a community where voluntary health communication among them has emerged and developed in the face of an urgent health crisis. This research provides evidence-based guidance for government health risk policy-makers and public managers on how mutual reinforcement between citizens’ participation in spontaneous risk discourses and the development of health-related behaviors in a community that faces the pandemic could function as a practical mechanism that facilitates risk information sharing and sustaining citizens’ commitment to protect their community from health risks [52]. That is, through health risk information-sharing ties that form in the face of the pandemic crisis, voluntary public health measures could be more acceptable and feasible and either better or poor subjective health conditions could be reinforced in a community. Considering this evidence, governments need to facilitate deliberation about health risk by providing detailed and timely pandemic information, promote the diffusion of recommended protective/preventive behaviors and make citizens voluntarily understand and accept them rather than just require citizens to change their health behaviors without risk discourses. For instance, utilizing multiple forms of social media channels where diverse citizens could easily answer questions and exchange up-to-date information could ensure adequate and timely risk communication. In contrast, for the emerging clusters of citizens whose mental disorders are deteriorating within their subgroups in a community, appropriate community- or governmental-level interventions and policy tools to respond to their mental health needs must be designed and implemented. People with serious mental health problems should be provided with specific hotlines or messaging services where they can receive customized information easily and feel cared for.

Despite the proposed theoretical foundations, the observed findings based on SAOM, and the resultant suggestions for health crisis management and policy in this study, there are several limitations to the current research design. First, the students’ network purposively selected as the boundary of network for this research is a subset of a broader pandemic communication network with numerous health risk information-seeking behaviors in a community. There may be additional socioeconomic and cultural attributive factors that influence these individual actors’ health communications and outcomes that are not controlled for. Furthermore, we also consider how any broader environmental factors and external events or shocks may influence the findings of the study.

## Figures and Tables

**Table 1:** Estimated Results for the Interactions between Risk Communication Networks and Health-related Behaviors across Three Subsequent Periods.

Variables	Coefficients	Standard Error
***Formation of Risk Communication Networks*** ***(Effects on Risk Communication Activity)*:**		
1. Rate of change from t_1_ to t_2_	4.40 ***	1.07
2. Rate of change from t_2_ to t_3_	3.76 ***	0.83
3. Out-degree (density)	−0.94 ***	0.35
4. Eligibility for risk communication 1 (effect of partner’s adoption of voluntary public health measures on link formation)	0.26 **	0.13
5. Entitlement to risk communication 1 (effect of one’s own adoption of voluntary public health measures on link formation)	0.35	0.32
6. Homophily 1 (partner selection based on similarity in voluntary adoption of public health measures)	2.43	1.90
7. Eligibility for risk communication 2 (effect of partner’s higher subjective health condition on link formation)	0.41	0.27
8. Entitlement to risk communication 2 (effect of one’s own higher subjective health condition on link formation)	0.22	0.29
9. Homophily 2 (partner selection based on similarity in subjective health)	−1.25 **	0.63
10. Reciprocity	1.54 ***	0.37
11. Transitive triplets	1.06 ***	0.22
12. In-degree popularity (sqrt)	−1.39 ***	0.49
13. Three cycles	−0.95 **	0.46
14. Same country	1.06 ***	0.22
15. Same gender	0.35	0.22
***Development of Health-Related Behaviors 1 (Effects on Voluntary Public Health Measures)*:**		
16. Rate of change from t_1_ to t_2_	0.77 ***	0.28
17. Rate of change from t_2_ to t_3_	2.81 **	1.38
18. Linear shape (tendency)	0.10	0.47
19. Quadratic shape (effect of voluntary public health measures on itself)	0.01	0.11
20. Effect of one’s own out-degree ties	−0.08	0.11
21. Effect of one’s own in-degree ties	0.16	0.28
22. Mutual influence (average similarity with partners)	0.35 ^**^	0.17
***Development of Health-Related Behaviors 2 (Effects on Subjective Health)*:**		
23. Rate of change from t_1_ to t_2_	1.98	1.37
24. Rate of change from t_2_ to t_3_	0.62 **	0.25
25. Linear shape (tendency)	−0.82	1.20
26. Quadratic shape (effect of subjective health conditions on itself)	−1.04	0.82
27. Effect of one’s own out-degree ties	−0.47	0.42
28. Effect of one’s own in-degree ties	0.84	0.93
29. Mutual influence (average similarity with partners)	0.65 *	0.34

*Note* 1: *** significant at the 1% level; ** significant at the 5% level; * significant at the 10% level. *Note* 2: The coefficients are from the standard Siena longitudinal analysis of directed network matrices that include 37 MPA students across the three time points. The overall maximum convergence ratio is 0.18, and all statistics converge with t-ratios close to zero (<0.10) with a minimum of 3000 iterations.

## References

[1] WHO (2020). Coronavirus Disease 2019 Situation Report-89.

[2] Cori L., Bianchi F., Cadum E., Anthonj C. (2020). Risk perception and COVID-19. Int. J. Environ. Res. Public Health.

[3] Lim S., Berry F.S., Lee K. (2016). Stakeholders in the same bed with different dreams: Semantic network analysis of issue interpretation in risk policy related to mad cow disease. J. Public Adm. Res. Theory.

[4] Allcott H., Boxell L., Conway J., Gentzkow M., Thaler M., Yang D.Y. (2020). Polarization and Public Health: Partisan Differences in Social Distancing during the Coronavirus Pandemic.

[5] Cheung H. Coronavirus: What Could the West Learn from Asia?. https://www.bbc.com/news/world-asia-51970379.

[6] Shaw R., Kim Y.-K., Hua J. (2020). Governance, technology and citizen behavior in pandemic: Lessons from COVID-19 in East Asia. Prog. Disaster Sci..

[7] Alarcon R.D. Mental health in a pandemic state: The route from social isolation to loneliness. https://www.psychiatrictimes.com/coronavirus/mental-health-pandemic-state-route-social-isolation-loneliness.

[8] Gabbatt A. Social recession: How isolation can affect physical and mental health. https://www.theguardian.com/world/2020/mar/18/coronavirus-isolation-social-recession-physical-mental-health.

[9] National Research Council (1989). Improving Risk Communication.

[10] National Research Council (1996). Understanding Risk: Informing Decisions in a Democratic Society.

[11] Reynolds B., Quinn S.C. (2008). Effective communication during an influenza pandemic: The value of using a crisis and emergency risk communication framework. Health Promot. Pract..

[12] Abrams E.M., Greenhawt M. (2020). Risk communication during COVID-19. J. Allergy Clin. Immunol. Pract..

[13] Husnayain A., Fuad A., Su E.C. (2020). Applications of google search trends for risk communication in infectious disease management: A case study of COVID-19 outbreak in Taiwan. Int. J. Infect. Dis..

[14] Freimuth V.S., Jamison A., Hancock G., Musa D., Hilyard K., Quinn S.C. (2017). The role of risk perception in flu vaccine behavior among African-American and white adults in the United States. Risk Anal..

[15] Jehn M., Kim Y., Bradley B., Lant T. (2011). Community knowledge, risk perception, and preparedness for the 2009 Influenza A/H1N1 Pandemic. J. Public Health Manag. Pract..

[16] Lee M., Ju Y., You M. (2019). The effects of social determinants on public health emergency preparedness mediated by health communication: The 2015 MERS outbreak in South Korea. Health Commun..

[17] Shalizi C.R., Thomas A.C. (2011). Homophily and contagion are generically confounded in observational social network studies. Sociol. Methods Res..

[18] Lim S., Nakazato H. (2019). Co-evolving supportive networks and perceived community resilience across disaster-damaged areas after the Great East Japan Earthquake: Selection, influence, or both?. J. Contin. Crisis Manag..

[19] McPherson J.M., Smith-Lovin L., Cook J.M. (2001). Birds of a feather. Annu. Rev. Sociol..

[20] Leenders R.T.A.J., Doreian P., Stokman F. (1997). Longitudinal behavior of network structure and actor attributes: Modeling interdependence of contagion and selection. Evolution of Social Networks.

[21] Smith K.P., Christakis N.A. (2008). Social networks and health. Annu. Rev. Sociol..

[22] Steglich C.E.G., Snijders T.A.B., Pearson M. (2010). Dynamic networks and behavior: Separating selection from influence. Sociol. Methodol..

[23] Basu A., Dutta M.J. (2008). The relationship between health information seeking and community participation: The roles of health information orientation and efficacy. Health Commun..

[24] Dutta-Bergman M.J. (2004). An alternative approach to social capital: Exploring the linkage between health consciousness and community participation. Health Commun..

[25] Pfeffer J., Salancik G.R. (1978). The External Control of Organizations.

[26] Ahn J., Chae D. (2019). The influences of socio-individual determinants and health information seeking on health-promoting behaviors among migrant women: A cross-sectional study. Jpn. J. Nurs. Sci..

[27] Griffin R.J., Yang Z., Huurne E.T., Boerner F., Ortiz S., Dunwoody S. (2008). After the flood: Anger, attribution, and the seeking of information. Sci. Commun..

[28] Lagoe C., Atkin D. (2015). Health anxiety in the digital age: An exploration of psychological determinants of online health information seeking. Comput. Hum. Behav..

[29] Centola D. (2011). An experimental study of homophily in the adoption of health behavior. Science.

[30] Crook B., Stephens K.K., Pastorek A.E., Mackert M., Donovan E.E. (2016). Sharing health information and influencing behavioral intentions: The role of health literacy, information overload, and the Internet in the diffusion of healthy heart information. Health Commun..

[31] Chuang Y., Huang Y., Tseng K., Yen C., Yang L. (2015). Social capital and health-protective behavior intentions in an influenza pandemic. PLoS ONE.

[32] Almedom A.M. (2005). Social capital and mental health. Soc. Sci. Med..

[33] Kim W., Kreps G.L., Shin C. (2015). The role of social support and social networks in health information-seeking behavior among Korean Americans. Int. J. Equity Health.

[34] Hanson-Easey S., Every D., Hansen A., Bi P. (2018). Risk communication for new and emerging communities: The contingent role of social capital. Int. J. Disaster Risk Reduct..

[35] Poortinga W. (2012). Community resilience and health: The role of bonding, bridging, and linking aspects of social capital. Health Place.

[36] Christakis N.A., Fowler J.H. (2013). Social contagion theory: Examining dynamic social networks and human behavior. Stat. Med..

[37] Eisenberg D., Golberstein E., Whitlock J.L., Downs M.F. (2013). Social contagion of mental health: Evidence from college roommates. Health Econ..

[38] Smith R.A., Zhu X., Shartle K., Glick L., M’ikanatha N.M. (2017). Understanding the public’s intentions to purchase and to persuade others to purchase antibiotic-free meat. Health Commun..

[39] Nakazato H., Lim S. (2016). Evolutionary process of social capital formation through community currency organizations: The Japanese case. VOLUNTAS Int. J. Volunt. Nonprofit Organ..

[40] Nakazato H., Lim S. (2017). Community rebuilding processes in a disaster-damaged area through community currency: The pilot project of “Domo” in Kamaishi, Japan. Disaster Prev. Manag..

[41] Snijders T.A.B., van de Bunt G.G., Steglich C.E.G. (2010). Introduction to actor-based models for network dynamics. Soc. Netw..

[42] Snijders T.A.B., Pickup M., Victor J.N., Montgomery A.H., Lubell M. (2018). Stochastic actor oriented models for network dynamics. The Oxford Handbook of Political Networks.

[43] Knoke D., Yang S. (2008). Social Network Analysis.

[44] Vaughan E., Tinker T. (2009). Effective health risk communication about pandemic influenza for vulnerable populations. Am. J. Public Health.

[45] Gomez O.A. (2013). Lessons from international students’ reaction to the 2011 Great East Japan Earthquake: The case of the School of Engineering at Tohoku University. Int. J. Disaster Risk Sci..

[46] Takashima M., Narita M., Oiwa Y., Abe A. Officials plead with young people to stop partying during pandemic. http://www.asahi.com/ajw/articles/13260124.

[47] Ministry of Health, Labour and Welfare (2020). About Coronavirus Disease 2019.

[48] Goldberg D.P., Williams P. (1988). A User’s Guide to the General Health Questionnaire.

[49] McDowell I. (2006). Measuring Health: A Guide to Rating Scales and Questionnaires.

[50] Goldberg D.P., Gater R., Sartorius N., Ustun T.B., Piccinelli M., Gureje O., Rutter C. (1997). The validity of two versions of the GHQ in the WHO study of mental illness in general health care. Psychol. Med..

[51] Saijo T. The Stay-at-home advisory as a hawk-dove game. https://www.tkfd.or.jp/en/research/detail.php?id=743.

[52] Jennings E.T., Hall J.L. (2012). Evidence-based practice and the use of information in state agency decision making. J. Public Adm. Res. Theory.

